# Availability and quality of anti-malarials among private sector outlets in Myanmar in 2012: results from a large, community-based, cross-sectional survey before a large-scale intervention

**DOI:** 10.1186/s12936-015-0778-0

**Published:** 2015-07-14

**Authors:** Hnin Su Su Khin, Ingrid Chen, Chris White, May Sudhinaraset, Willi McFarland, Megan Littrell, Dominic Montagu, Tin Aung

**Affiliations:** Population Services International Myanmar, No. 16, Shwe Gon Taing Street 4, Yangon, Myanmar; Global Health Sciences, University of California, San Francisco, 550 16th Street, 3rd Floor, San Francisco, CA 94158 USA; Population Services International, 1120 19th St NW Suite 600, Washington, DC 20036 USA

**Keywords:** Malaria, Artemisinin-based monotherapy, Drug resistance, ACTwatch, Private sector, Myanmar

## Abstract

**Background:**

Global malaria control efforts are threatened by the spread and emergence of artemisinin-resistant *Plasmodium falciparum* parasites. In 2012, the widespread sale of partial courses of artemisinin-based monotherapy was suspected to take place in the highly accessed, weakly regulated private sector in Myanmar, posing potentially major threats to drug resistance. This study investigated the presence of artemisinin-based monotherapies in the Myanmar private sector, particularly as partial courses of therapy, to inform the targeting of future interventions to stop artemisinin resistance.

**Methods:**

A large cross-sectional survey comprised of a screening questionnaire was conducted across 26 townships in Myanmar between March and May, 2012. For outlets that stocked anti-malarials at the time of survey, a stock audit was conducted, and for outlets that stocked anti-malarials within 3 months of the survey, a provider survey was conducted.

**Results:**

A total of 3,658 outlets were screened, 83% were retailers (pharmacies, itinerant drug vendors and general retailers) and 17% were healthcare providers (private facilities and health workers). Of the 3,658 outlets screened, 1,359 outlets (32%) stocked at least one anti-malarial at the time of study. Oral artemisinin-based monotherapy comprised of 33% of self-reported anti-malarials dispensing volumes found. The vast majority of artemisinin-based monotherapy was sold by retailers, where 63% confirmed that they sold partial courses of therapy by cutting blister packets. Very few retailers (5%) had malaria rapid diagnostic tests available, and quality-assured artemisinin-based combination therapy was virtually nonexistent among retailers.

**Conclusion:**

Informal private pharmacies, itinerant drug vendors and general retailers should be targeted for interventions to improve malaria treatment practices in Myanmar, particularly those that threaten the emergence and spread of artemisinin resistance.

## Background

In Southeast Asia, drug resistance of *Plasmodium falciparum* parasites against first-line anti-malarials, the artemisinins, threaten malaria control efforts worldwide. Much like the previous spread of chloroquine resistance, the first signs of artemisinin-resistant parasites were seen in Cambodia in 2009 [1], which have since emerged independently in several locations and have now spread to Myanmar, Cambodia, Laos, Thailand, and Vietnam [2–5]. Myanmar accounts for approximately 85% of the reported malaria cases in the Greater Mekong sub-region of Asia [2], and the spread of artemisinin resistance throughout Myanmar would be devastating not only for the 37 million individuals living in Myanmar’s malaria-endemic areas, but for the global fight against malaria.

In Myanmar, malaria is a well-known problem having been a leading cause of morbidity and mortality in Myanmar for over two decades [2]. Although malaria endemicity in Myanmar has been dropping in recent years, with an estimated 495,000 cases in 2013 [2], the spread of drug-resistant *P. falciparum* parasites threatens these gains [5]. To prevent a global health emergency, all practices that threaten the emergence of *P. falciparum* parasites must be changed, and all artemisinin-resistant parasites must be contained and eliminated [6, 7].

In Myanmar, the informal private sector has been implicated for practices that threaten the emergence of drug resistance. When this study was initiated in 2012, it was suspected that the most common practice in Myanmar was to treat symptoms of malaria with an incomplete course of artemisinin-based monotherapy. This practice was largely implicated in the emergence of drug resistance, which may be selected for when parasites survive low, subtherapeutic doses of drug exposures [7]. To prevent drug resistance against artemisinin-class drugs, the World Heath Organisation (WHO) recommends the use of artemisinin-based combination therapy (ACT) as a first-line treatment for *P. falciparum* malaria [5]. However, ACT is expensive, and when available in Myanmar, patients often could not afford it. In this case, providers were suspected to cut the blister packets of ACT, selling partial courses of artemisinin-based monotherapy instead [7].

In order to remove artemisinin monotherapies from the market in Myanmar, and to encourage the sale of only full courses of ACT, there was first a need determine where artemisinin-based monotherapies were sold, and whether they were indeed sold as partial courses of therapy. This study targeted the private sector, the highly accessed, weakly regulated, first point of care of fever for most individuals in Myanmar where the widespread sale of artemisinin-based monotherapy was suspected to take place [2]. The private sector is comprised of private clinics, pharmacies, informal drug shops, and general retailers, where antimalarial drugs and rapid diagnostic tests (RDTs) for malaria can be sold by any individual, without the need for certification or formal training.

This study, conducted in Myanmar in 2012, aimed to determine what types of private outlets exist, what types of anti-malarial drugs were in stock at each outlet, whether RDTs for malaria were available, and whether providers were selling partial courses of treatment. The information gained from this study has since been used to enable a large-scale transformation of the anti-malarial market in Myanmar, where artemisinin-based monotherapies have been successfully replaced with ACT [8]. This study should be used as an example of a baseline assessment for other countries seeking to improve malaria treatment and diagnostic practices in the private sector.

## Methods

### Study area and design

**Figure 1:**
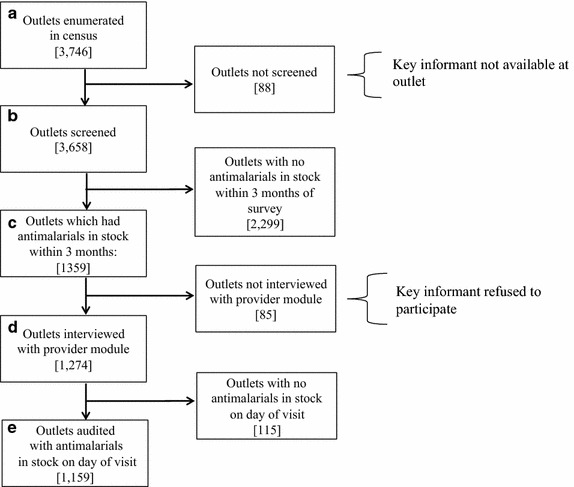
Selection process for outlet inclusion.

The study employed a cross-sectional survey comprising a full census of 3,746 outlets across 26 townships in Myanmar (the country contains a total of 325 townships), carried out between March and May 2012 (Figure 1). The methods used for this study were adapted from the ACTwatch programme [9], which recorded the types of private outlets sampled, outlet locations, availability of various anti-malarials among outlets sampled, provider demographics, and a brief assessment of provider knowledge and practices for malaria treatment.

### Sampling approach

A cluster sampling approach was adopted using a two-stage probability proportion to size (PPS) cluster design, with the measure of size being the relative cluster population. Clusters were defined as geographical areas, such as wards in urban areas and village tracts in rural areas. Sampling frames of all townships were developed after excluding 51 townships that were inaccessible for security reasons and/or inaccessibility.

At the first stage of sampling, 26 townships across Myanmar were selected using PPS. The number of townships included was based on operational feasibility. In each selected township, the sampling frame consisted of wards (which are the smallest administrative geographic unit to represent an urban area) and village tracts. At the second stage of sampling, a total of 122 wards and 130 village tracts were selected using PPS. Each selected township had at least four wards and five village tracts. For each township, five wards and five village tracts were selected using PPS unless the selected township had only four wards, in which case all four wards were selected. Within the selected ward or village tract, a census of all outlets in the private sector that could potentially sell or provide anti-malarials to individuals was conducted using a snowball sampling approach. Population Services International (PSI) field staff members started either from the highway bus terminal or from market places and walked to nearby general stores or drug shops, asking individuals working in shops whether they knew of other places in the ward or village selling or providing anti-malarial treatments. Maps were used to help to identify cluster boundaries and the areas that PSI field staff members would visit. Each provider interviewed would also be asked to identify other outlets providing anti-malarial treatments in the area.

A total of 3,746 outlets were identified (Figure 1a), and key informants were available in 3,658 of these outlets (Figure 1b). Of these 3,658 outlets, key informants in 1,359 outlets reported that they stocked anti-malarials within 3 months of the survey date (Figure 1c), were asked to participate in the provider module, and 1,274 outlets participated [85 outlets (6%) declined to participate] (Figure 1d). Of these 1,274 outlets, 1,159 key informants in outlets reported that that they stocked anti-malarials on the day of the survey visit, all of whom participated in a full anti-malarial drug audit (Figure 1e). The analysis of anti-malarial stocks and provider knowledge in this report is based on these 1,159 outlets.

### Inclusion criteria

An outlet was defined as any point of sale or provision of commodities for individuals. Two types of outlets were sampled; healthcare providers and retailers. Healthcare providers were comprised of privately owned hospitals or clinics tended by general practitioners (which may or may not be affiliated with a franchise network), as well as community health workers providing treatment outside of the public health facilities. Retailers were comprised of registered pharmacies, itinerant drug vendors (hawkers), and general retailers such as village stores, groceries, and general stores. In Myanmar, registration with the City Development Committee is required to open a pharmacy, also called a ‘drug shop’, and defined as any outlet that mainly sells drugs. However, as regulation and enforcement are weak in Myanmar, many pharmacies in rural areas like the ones studied herein do not seek registration. The majority of itinerant drug vendors are mobile drug vendors that serve people within a catchment area of approximately five or six villages within their area of residence. Most itinerant drug vendors received training from previous jobs (e.g. assistant to an army nurse, a nurse aid at a private hospital, or a clinical assistant to a medical doctor) and provide services in the same place for many years, and are therefore well-known by the population, and easy to find and identify. For all outlets included, a key informant, defined as the owner of the shop, provider or pharmacist working at the shop, needed to be available at the time of survey. All key informants were required to be at least 18 years of age, and willing to provide informed verbal consent to participate in the study.

### Data collection instruments

The outlet questionnaire was comprised of three modules: (1) a screening module for all outlets included; (2) a provider module for all outlets that reported to stock anti-malarials in the past 3 months; and (3) an audit module for outlets with anti-malarials in stock on the day of interview.

The screening module was used to record the type and location of all outlets, and to identify outlets in which to conduct the provider module, and the audit module. The provider module was used to collect descriptive information on providers, including basic demographics such as the level of education and type of formal health training received by all employees at the outlet, as reported by the provider interviewed. This module also asked providers whether they could name different types of malaria (*P. falciparum* vs *Plasmodium vivax* malaria), whether they knew the government recommended treatment for uncomplicated *P. falciparum* malaria (the use of the ACT artemether–lumefantrine), and whether they ever cut blister packs, or sold partial courses of anti-malarials over the past month, as well as their reasons for doing so.

The audit module was based on standard methods developed and tested by the WHO Health Action International questionnaire for essential medicines [10]. For each anti-malarial drug in stock, the interviewer asked to see the drugs, and recorded information on the brand and generic names, drug strength, package size, country of origin, and expiration date (from anti-malarial packaging). The interviewer then asked the provider to recall the price to the customer, and the amount sold in the last week (from records where available). Artemisinin-based monotherapy was defined as an anti-malarial medicine that has a single active compound called artemisinin or one of its derivatives, including artesunate and artemether. Non-artemisinin therapy included chloroquine, quinine and primaquine, which is recommended for the treatment of vivax malaria. Drugs intended solely for malaria chemoprophylaxis were not included in this study. The audit module also collected information on whether malaria rapid diagnostic test (RDTs) were in stock on the day of the interview.

### Data collection procedures

Paper questionnaires were administered during data collection. The questionnaire was finalized in English and translated and administered in local Myanmar language versions. All staff involved in the study, including the data collection team, underwent a 6-day training on how to identify anti-malarial medicines, including the differences between ACT and non-ACT, brands and generic names, drug packaging, and drug strength. The training addressed the purpose of the study, the importance of consent, and how to administer the consent form and study questionnaires. The study received ethical approval from the PSI Research Ethics Board.

### Data analysis

To allow for meaningful comparisons between anti-malarials with different treatment courses, the volume of anti-malarials recorded in the audit module was standardized using the adult equivalent treatment dose (AETD). One AETD was defined as the amount of the drug, in milligrams, that a 60 kg adult would need in order to receive a full course of treatment according to the availability of information according to the following three sources of information, in order of priority: (1) the WHO; (2) peer reviewed literature; or (3) from manufacturer guidelines. The provider-reported number of packages distributed was scaled to the equivalent number of AETD courses sold in the previous week. For combination anti-malarials, one drug in the combination was selected for these calculations. For ACT, this was always the artemisinin-derivative component (e.g., the artemether component of artemether–lumefantrine).

For the purpose of this study, anti-malarials were analyzed as three policy-relevant categories: ACT, artemisinin-based monotherapy, and non-artemisinin therapy. ACT was sub-divided into quality assured ACT (QA-ACT), defined as an ACT pre-qualified by WHO and/or authorized for marketing by a Stringent Drug Regulatory Authority [11], and non-quality assured ACT (non QA-ACT), defined as any ACT not included in the WHO prequalification list. Artemisinin-based monotherapy was sub-categorized into oral artemisinin-based monotherapy, a contributing factor for drug resistance malaria [2], and injectable artemisinin-based monotherapy, the recommended drug for severe malaria in hospital settings [12].

In order to test for statistically significant differences between healthcare providers and retailers (at the 0.05 level), t tests were used for continuous variables, and Chi square tests for categorical variables.

To ensure data quality, two-pass verification, also known as double data entry was conducted using a database system designed with in-built checks for consistency and range values. Data analysis was conducted in Stata 11.0 (Stata Corp College Station, TX, USA), which included descriptive summaries across different outlet types. Sampling weights based on the cluster population size used allow for the provision of national estimates.

## Results

### Outlet screening

Of the 3,658 outlets screened, 83% were retailers and 17% were healthcare providers (Table 1). A total of 46% of the outlets were located in urban areas, and 54% were in rural areas. In urban areas, private facilities (88%) and pharmacies (86%) were the most commonly found drug providers, while in rural areas, health workers (79%) and itinerant drug vendors (80%) were the most commonly found drug providers. General retailers were more evenly distributed between rural and urban areas, with 60% being located in rural areas and 40% in urban areas.

**Table 1:** Demographics of private sector outlets screened

	Total	Healthcare providers			Retailers				*p* value*
		Private facility	Health worker	Total	Pharmacy	Itinerant drug vendor	General retailer	Total	
N (%)	3,658	283 (8%)	324 (9%)	607 (17%)	450 (12%)	304 (8%)	2,297 (63%)	3,051 (83%)	
Location									
Urban	1,684 (46%)	249 (88%)	68 (21%)	317 (51%)	387 (86%)	61 (20%)	919 (40%)	1,367 (45%)	<0.05
Rural	1,974 (54%)	34 (12%)	256 (79%)	290 (49%)	63 (14%)	243 (80%)	1,378 (60%)	1,684 (55%)	
Stocking any anti-malarial at time of survey	1,159 (32%)	194 (69%)	218 (67%)	412 (68%)	342 (76%)	136 (45%)	269 (12%)	747 (24%)	<0.001

* Comparing healthcare providers to retailers.

A total of 1,159 outlets screened (32%) stocked at least one anti-malarial at the time of study (Table 1). Anti-malarial availability was highest in pharmacies (76%), followed by private facilities (69%) and health workers (67%), and lowest among general retailers (12%), the most common outlet type screened (2,297 outlets, 63%).

### Stock audit module

Among outlets that had anti-malarials on the day of the survey, 58% of outlets stocked oral artemisinin-based monotherapy, and 24% stocked QA-ACT (Table 2). QA-ACT was available in 60% of treatment professional outlets, and 4% of retailer outlets (*p* < 0.001). Among retailers, QA-ACT was lowest among pharmacies (3%) and general retailers (4%). Artemisinin-based monotherapies were present in 74% of retailer outlets with the highest percentages seen among pharmacies (89%) and general retailers (77%), as compared to only 3% of treatment professional facilities (*p* < 0.001). The presence of injectable artemisinin monotherapies was similar between healthcare providers and retailer outlets (33 and 28%, respectively). The availability of non-artemisinin drugs was highest among itinerant drug vendors (83%), and otherwise similar across all other outlet types (49–64%). RDTs were available in 62% of treatment professional outlets, and only 5% of retailers outlets (*p* < 0.001), with lowest rates (2%) among general retailers.

**Table 2:** Availability of anti-malarial drugs and malaria rapid diagnostic tests in private outlets

	Total	Healthcare providers			Retailers				*p* value**
		Private facility	Health worker	Total	Pharmacy	Itinerant drug vendor	General retailer	Total	
N (%)	1,159	194 (17%)	218 (19%)	412 (36%)	342 (29%)	136 (12%)	269 (23%)	747 (64%)	
Availability of anti-malarial									
Quality assured ACT^†^	280 (24%)	93 (48%)	156 (72%)	249 (60%)	10 (3%)	11 (8%)	10 (4%)	31 (4%)	<0.001
Non-artemisinin drugs	677 (58%)	118 (61%)	140 (64%)	258 (62%)	168 (49%)	113 (83%)	138 (51%)	419 (56%)	<0.05
Injectable artemisinin-based monotherapy	341 (29%)	76 (39%)	59 (27%)	135 (33%)	153 (45%)	33 (24%)	20 (7%)	206 (28%)	<0.001
Oral artemisinin-based monotherapy	677 (58%)	83 (43%)	44 (20%)	127 (31%)	304 (89%)	39 (29%)	207 (77%)	550 (74%)	<0.001
Non QA-ACT	67 (6%)	24 (12%)	2 (1%)	26 (6%)	38 (11%)	1 (1%)	2 (1%)	41 (6%)	<0.05
Availability of RDT	293 (25%)	99 (51%)	157 (72%)	256 (62%)	20 (6%)	12 (9%)	5 (2%)	37 (5%)	<0.001

Among those outlets that had any anti-malarials in stock on the day of visit.

** Comparing healthcare providers to retailers.

^†^Columns do not always add up to 100% because one outlet can stock more than one type of anti-malarial drug; percentages are column percent.

**Figure 2:**
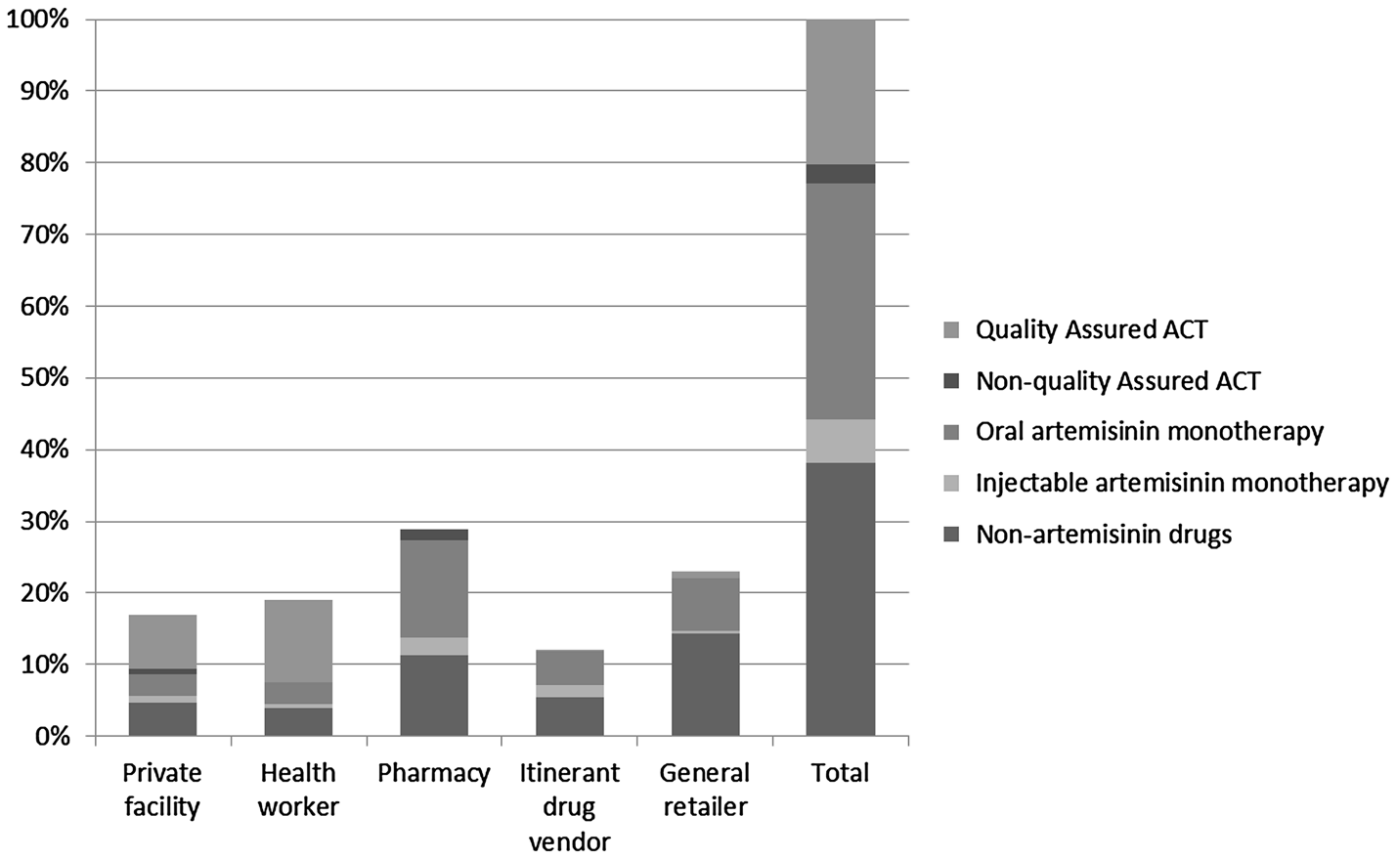
Relative volume of anti-malarial drugs sold in the previous 7 days by outlet type in Myanmar.

Estimates of anti-malarials volumes sold within the past 7 days showed the highest rate of sales for non-artemisinin drugs (38.1%), followed by oral artemisinin-based monotherapy (32.9%) and QA-ACT (21.7%) (Figure 2). Analysis across different outlet types showed that pharmacies were the most significant contributor of oral artemisinin-based monotherapies. QA-ACT was virtually non-existent among all retailers, but was widely available among healthcare providers.

### Provider module

Among outlets surveyed, 55% were staffed by university graduates, 38% by high school graduates, and 7% having completed primary school as their highest level of education (Table 3). The attainment of a University degree was higher among healthcare providers (73%) than retailers (45%, *p *< 0.001), with highest levels seen among private facilities (99%) and lowest levels seen among itinerant drug vendors (19%).

**Table 3:** Provider attributes and blister-cutting practices in private sector outlets stocking anti-malarials

	Total	Healthcare providers			Retailers				*p* value*
		Private facility	Health worker	Total	Pharmacy	Itinerant drug vendor	General retailer	Total	
N (%)	1,159	194 (17%)	218 (19%)	412 (36%)	342 (29%)	136 (12%)	269 (23%)	747 (64%)	
Education									
Primary	81 (7%)	0 (0%)	4 (2%)	4 (1%)	4 (1%)	16 (12%)	57 (21%)	77 (10%)	<0.001
High school	446 (38%)	2 (1%)	107 (49%)	109 (26%)	96 (28%)	94 (69%)	147 (55%)	337 (45%)	<0.001
Graduate	632 (55%)	192 (99%)	107 (49%)	299 (73%)	242 (71%)	26 (19%)	65 (24%)	333 (45%)	<0.001
Type of health provider^†^									
Medical doctor	185 (16%)	178 (92%)	0 (0%)	178 (43%)	7 (2%)	0 (0%)	0 (0%)	7 (1%)	<0.001
Nurse/midwife	229 (20%)	21 (11%)	122 (56%)	143 (35%)	52 (15%)	26 (19%)	8 (3%)	86 (12%)	<0.001
Health assistant	26 (2%)	2 (1%)	20 (9%)	22 (5%)	3 (1%)	1 (1%)	0 (0%)	4 (1%)	< 0.05
Pharmacist	35 (3%)	5 (3%)	2 (1%)	7 (2%)	24 (7%)	1 (1%)	3 (1%)	28 (4%)	<0.001
Knows types of malaria	523 (45%)	97 (50%)	192 (88%)	289 (87%)	116 (34%)	64 (47%)	54 (20%)	234 (31%)	<0.001
Know first-line treatment for falciparum malaria	233 (20%)	95 (49%)	113 (52%)	208 (50%)	14 (4%)	11 (8%)	5 (2%)	30 (4%)	<0.001
Provider practice: do cut the blisters	531 (46%)	19 (10%)	43 (20%)	62 (15%)	205 (60%)	57 (42%)	207 (77%)	469 (63%)	<0.000

* Comparing healthcare providers to retailers.

^†^Columns do not always add up to 100% because multiple responses are possible.

Formal health training among outlets surveyed showed that 16% of outlets were operated or staffed by medical doctors, 20% by nurse or midwives, 2% by health assistants, and 3% by pharmacists, with much higher rates of formal training among healthcare providers (43% were medical doctors and 33% were nurses/midwives) than retailers (1% were medical doctors and 12% were nurses/midwives, *p* < 0.001).

A brief assessment of provider knowledge of malaria revealed that 45% of providers could name *P. falciparum* and *P. vivax* as different types of malaria, and only 20% of providers were aware that artemether–lumefantrine was the recommended first-line treatment of malaria (Table 3). Within these individuals, correctly answered questions were higher among healthcare providers than retailers (for types of malaria, 87 vs 31%, *p* < 0.001, for the first-line treatment of malaria, 50 vs 4%,* p *< 0.001), with lowest levels observed among general retailers (20% for types of malaria, and 2% for first-line treatment).

A total of 46% of the 1,159 outlets that stocked anti-malarial drug(s) at the time of survey reported that they cut blister packets and/or sold partial courses of anti-malarial treatments to patients (Table 2). This practice was reported by 63% of retailers and 15% of healthcare providers (*p* < 0.001), with highest rates reported by general retailers (77%). The most common reason that providers stated for cutting blister packets was due to customer request (68%), and the second most common reason was that customers could not afford a full blister or pack of treatment (25%). Other reasons also included that providers stated that cutting a blister packet made it easier for patients to take medicine (17%), or that cutting blister packets provided sufficient treatment to patients (15%).

## Discussion

This study was the first to investigate the private sector anti-malarial market in Myanmar on a large scale, examining the availability of anti-malarial drugs and malaria RDTs in private outlets, the types of anti-malarial drugs available in various types of outlets, provider demographics and basic knowledge of types of malaria and treatment guidelines for falciparum malaria, and provider practices pertaining to the cutting of blister packets and/or the same of partial courses of anti-malarial drugs.

This study confirmed that oral artemisinin-based monotherapy was indeed widely available in the Myanmar private sector, and furthermore showed that within the private sector, retailers should take priority in receiving interventions designed to improve malaria treatment and diagnosis practices. The presence of oral artemisinin-based monotherapy was much higher among retailers (74%) than healthcare providers (31%), and retailers reported higher rates of cutting blister packs (63%) than healthcare providers (15%). Education levels and provider knowledge on the types of malaria and treatment guidelines for falciparum malaria were also much lower among retailers compared to healthcare providers (45 vs 73% university graduates, 31 vs 76% named types of malaria correctly, and 4 vs 52% knew the first-line treatment for falciparum malaria, respectively). QA-ACT was also virtually non-existent among retailers (4%), but found in 60% of healthcare provider outlets.

These results suggest two types of interventions to improve malaria treatment and diagnostic practices within the private sector. The first is the targeting of retailers to replace the sale of oral artemisinin-based monotherapy with QA-ACT. This could minimize practices suspected to contribute to the development of artemisinin resistance, as QA-ACT contains a long-acting partner drug designed to clear remaining malaria parasites when levels of short-acting artemisinin-class drugs are in the body. Since under-dosing of artemisinin-class can contribute to the emergence of drug resistance, the replacement of QA-ACT with oral artemisinin-based monotherapy can minimize risks that parasites develop artemisinin resistance [7]. Since QA-ACT have higher costs than oral artemisinin-based monotherapy [13], selling them at a subsidized cost is expected to be necessary for intervention effectiveness [7].

The second type of intervention is to provide education to providers, once again targeting retailers, who had lower levels of education than healthcare providers, and cut blister packets more often. Education should be focused on improved malaria treatment, including the use of QA-ACT as per treatment guidelines for falciparum malaria, as well as the dangers of cutting blister packets in case it should lead to under-dosing, and hence increased risks of drug resistance. This study did not assess whether drugs were dispensed at accurate doses, and did not assess whether providers knew of proper dosing guidelines, so future studies or educational interventions should ensure that providers are indeed aware of proper dosing practices. Furthermore, it should also be emphasized that while retailers should be prioritize for interventions, healthcare providers showed limited knowledge on the first-line treatment of falciparum malaria (50%), and could also therefore benefit from interventions.

The presence of injectable artemisinin-based monotherapies in private outlets (33% among healthcare providers, 28% among retailers) could also warrant further investigation, because injectable artesuate is recommended for the treatment of severe malaria [12]. Future studies can investigate whether individuals with severe malaria indeed present in private facilities, and if so, whether providers are able to provide proper care for severe malaria, an assessment which should include the ability of providers to administer injections, and whether they have access to clean needles and are able to safely dispose of used ones.

After this study took place, the two interventions suggested above were indeed carried out, to successfully replace oral artemisinin-based monotherapies with subsidized QA-ACT [8], and then to ensure their proper use through the use of subsidized RDTs [14–16]. Interestingly, educational campaigns to both the provider and the community proved to be effective, with increased educational visits to providers leading to increased levels of RDT use [14–16]. This follow-up intervention also offered the potential to crowd out the supply of sub-standard and counterfeit ACT, offering a relatively simple solution to an otherwise challenging problem [7].

This study offers methods that can be used by other countries seeking to transform malaria diagnosis and treatment practices in the private sector. Indeed, ACTWatch methods have been used in several countries in Africa [8, 17], and Cambodia has also undertaken a nationwide landscaping study on the availability of anti-malarials within the private sector [18]. This type of large-scale cross sectional study provides baseline information on what kind of providers exist in the private sector, what types of drugs they stock, and which types of interventions are likely to improve the quality of the care offered. A number of factors must be considered in local contexts, both with regard to interpreting results, and navigating future interventions. The successful follow-up intervention to this study in Myanmar is partially attributed to the ease of permeating the supply chain, as Myanmar providers rely on a highly centralized supply chain offered by the AA pharmacy. The broader notion however, that the private sector can be engaged with ACT subsidies, is entirely transferable as long as the actual strategies used follow local cultural contexts.

## Limitations

The study also did not access public facilities in Myanmar and therefore did not provide the landscape of the overall anti-malarial market in areas studied. Although the size of the study allowed for the provision of national estimates, it is possible that this study was not nationally representative, as townships excluded for security reasons may have different accessibility to anti-malarial drugs. Snowball sampling, the method used to identify private providers in this study, has inherent limitations including that the researcher relies mainly on previous subjects observes such that there is potential for sampling bias as participants are more likely to nominate someone who has similar characteristics to themselves. As a result of these potential biases, the representativeness of the sample is not guaranteed, as the researcher cannot be sure that they have indeed obtained a true distribution of private providers throughout the population. The volumes of anti-malarials documented in this study were also self-reported by providers, and therefore subject to recall biases, as well as a potential desire for a shopkeeper to tell the interviewer what they wanted to hear. The study also did not account for all potential risk factors contributing to the selection for drug resistance (e.g. patient adherence to antimalarial drugs). The study did not assess the actual dispensing of drugs and the accuracy of doses dispensed, so the provider reporting of cutting blister packets does not necessarily imply under-dosing. Finally, although practices implicated in the spread of artemisinin drug resistance were identified, there was no proven causal relationship.

## Conclusion

Oral artemisinin-based monotherapy was most commonly found among general retailers in rural settings, and these providers commonly cut blister packs when selling anti-malarials to patients. As a result of this study, general retailers were targeted for a large-scale intervention to replace artemisinin-based monotherapy with subsidized QA-ACT throughout the Myanmar private sector. This study should be used as an example of a baseline assessment to improve malaria treatment practices in Myanmar in locations where the private sector is a common first point of care for fever.
